# Protracted maternal malnutrition induces aberrant changes in maternal uterine artery hemodynamics and the metabolic profiles of the dam and neonate

**DOI:** 10.3389/fphys.2024.1501309

**Published:** 2024-12-05

**Authors:** Jennifer F. Thorson, Ligia D. Prezotto

**Affiliations:** ^1^ Nutrition, Growth and Physiology Research Unit, U.S. Meat Animal Research Center, USDA, Agricultural Research Service, Clay Center, NE, United States; ^2^ Physiology Laboratory, Department of Animal Science, University of Nebraska-Lincoln, Lincoln, NE, United States

**Keywords:** blood flow, bovine, cerebrospinal fluid, gestation, metabolite, neonate, nutrition

## Abstract

Malnutritional stress during gestation is a well-established driver of metabolic disfunction in offspring. Extended exposure to malnutrition requires metabolic plasticity as the animal shifts toward a catabolic state. In this paper we demonstrate the influence of malnutrition throughout gestation on uterine artery hemodynamics and the metabolism of the dam and neonate. We hypothesized that gestational malnutrition reduces blood flow of the maternal uterine artery and regulates the metabolic profile of the dam and offspring. Further, the combination of these factors consequently influences the concentration of metabolites in the cerebrospinal fluid of the neonate at birth. To test our hypotheses, pregnant cows caring a single female fetus were assigned to treatments by age and body condition score to one of three individually-fed dietary treatments: Underfed, Control, or Overfed throughout gestation. Uterine blood flow was measured via transrectal Doppler ultrasonography in late gestation. Blood samples were collected from dams throughout gestation, and blood and cerebrospinal fluid were collected from neonates at birth to analyze concentration of metabolites. In the current report, we reveal that maternal malnutrition regulates uterine artery hemodynamics and the maternal metabolic profile throughout gestation. This is the first report to demonstrate that maternal undernutrition leads to an increase in the concentration of urea nitrogen in neonates. Finally, a concentration gradient of metabolites from the dam to neonatal cerebrospinal fluid was observed, which may have potential implications for central nervous system development. These findings not only illustrate the complexity of the maternal-to-fetal interaction required to support the growth of the fetus and homeostasis of the dam but also reveals a novel avenue for investigating the influence of protracted maternal malnutrition on metabolic pathway preferences in offspring. Moreover, these findings are of paramount importance in the development of intervention strategies for morbid neonates.

## 1 Introduction

The maternal nutritional status maintained during pregnancy affects not only the dam but also largely influences fetal development, as previously reviewed (Hales and Barker, 2001; Wu et al., 2006). The level of nutrition the dam receives influences the maternal uterine environment, which determines the rate of fetal development (Wallace et al., 2002). Mechanisms that affect fetal development include placental functional capacity, transference of nutrients and oxygen via uteroplacental pathways, nutrient availability to the fetus, and preferential metabolic pathways (Bell and Ehrhardt, 2002; Redmer et al., 2004; Fowden et al., 2005; Reynolds et al., 2006). Metabolites from the dam are transferred to the fetus during gestation, including beta-hydroxybutyrate (BHB), non-esterified fatty acids (NEFA), glucose, and urea nitrogen (UN) (Kleeman et al., 1962; Banks et al., 1997; Devraj et al., 2011; Shippy et al., 2020). Regulated transport of these metabolites across the fetal blood–brain barrier is critical for normal neuronal development and function. However, no reports have assessed the influence of maternal nutrition on the metabolite profile of the cerebrospinal fluid (CSF) in the neonate.

Maternal nutritional stress during gestation has been well established as a main driver of the development of metabolic disease in offspring. Impaired glucose tolerance is correlated with increased blood pressure and disturbed lipoprotein metabolism in offspring exposed to an adverse uterine environment (Barker et al., 1993). Maternal protein consumption during gestation has also been manipulated to assess the effect on fetal development and offspring health during postnatal life; however, mixed results have been reported, showing both negative and positive aspects of said alterations (Summers et al., 2015; Nascimento et al., 2022). Nutritional challenges associated with pregnancy also alter the concentrations of fatty acids that influence the programing of fetal metabolism linked to metabolic diseases such as insulin resistance and modifications in the hypothalamic appetite and reward systems (Hsu and Tain, 2019; Morris, 2009). Furthermore, the regulation of postnatal energy perception involves an integrated regulation between the brain and peripheral hormones and metabolites (Chaudhri et al., 2006; Karisa et al., 2014) that may also contribute to subfertility in offspring.

Other researchers have investigated the pattern of uterine blood flow in response to diet and/or gestational period in dairy and beef cows (Herzog et al., 2011; Camacho et al., 2014). However, no study has previously investigated the concurrent effects of over- and undernutrition throughout the entire gestational period. Furthermore, no reports have investigated the effect of maternal malnutrition on the neonatal metabolic profile within the peripheral circulation and CSF. Therefore, the aim of the current research is to not only understand how maternal malnutrition affects uterine artery hemodynamics but also assess metabolite patterns in the dam throughout gestation and the neonate at birth to assess the nutritional quality of the blood delivered *in utero* that ultimately regulates the development of the central nervous system.

## 2 Materials and methods

Experiments were conducted in accordance with the Guide for the Care and Use of Agricultural Animals in Agricultural Research and Teaching (FASS, 2010) and approved by the Montana State University Agricultural Animal Care and Use Committee.

### 2.1 Experimental design

At least 28 days prior to breeding, multiparous Angus cows (4–6 years of age; body condition score, 5.5–6.5) were trained to use an individual feed bunk (Calan Broadbent Feeding System; American Calan; Northwood, NH, United States) and acclimated to a diet consisting of chopped grass hay, straw pellet, and a protein pellet fortified with vitamins and minerals to meet or exceed dietary recommendations (BCNR, 2016). Estrus was synchronized, and the cows were bred by artificial insemination with sex-sorted semen from a single Angus sire. Singleton pregnancies were confirmed 28 days post-breeding via ultrasonography. Pregnant cows carrying a single fetus were assigned to one of three individually fed dietary treatments—Underfed (n = 6), Control (n = 5), or Overfed (n = 6)—based on age and body condition score. Underfed, control, and overfed diets were offered for the remainder of gestation (28 to approximately 280 days) at 60%, 100%, and 140% of nutrient recommendations for cows during gestation (BCNR, 2016), respectively. The dietary treatments were selected for their ability to induce differences in the maternal body condition while also reflecting typical changes observed in production due to environmentally regulated nutrient supply. The cows were fed a combination of chopped grass hay and straw pellet at 60, 100, or 140% of their dietary net energy requirements and a protein pellet fortified with minerals and vitamins to meet or exceed metabolizable protein, mineral, and vitamin recommendations for all cows by stage of gestation (BCNR, 2016). The dietary intake was adjusted every 28 days to meet the targeted body weight change and changes in net energy requirements for the stage of gestation. Fetal sex was confirmed 56 days post-breeding via ultrasonography. Pregnant cows carrying a male fetus were excluded from the experiment (n = 2 Underfed; n = 1 Control; and n = 0 Overfed), resulting in n = 4, 4, and 6 for Underfed, Control, and Overfed treatments, respectively.

### 2.2 Harvest of plasma and cerebrospinal fluid

Maternal blood samples were collected following a 12-h nocturnal feed withdrawal period. Blood samples were collected every 28 days throughout gestation, beginning on the day of breeding (day 0) and continuing until day 280 of gestation or parturition, whichever occurred first. If parturition occurred prior to day 280, the cows were bled the day after parturition to allow for blood collection following feed withdrawal, comparable to that harvested during gestation. At parturition, the offspring were removed from the dam prior to nursing, and neonatal blood and CSF samples were collected within 1 h of birth. The blood samples were collected in evacuated tubes containing EDTA for the collection of plasma, and the tubes were placed immediately on ice for 30 min. Cerebrospinal fluid samples were collected, as previously described by Braun & Attiger (2016), using an ultrasound guide with a 20-gauge atraumatic spinal needle attached to a 3-mL syringe. The cerebrospinal fluid was then transferred to microcentrifuge tubes and immediately placed on ice. The samples were centrifuged at 2,000 × g for 15 min, plasma or red blood cell-free CSF was harvested, and samples were stored at −80°C until analysis. Cerebrospinal fluid was centrifuged to ensure that blood contamination did not occur and maintain consistent handling between all samples.

### 2.3 Metabolite assays

The plasma and CSF samples were analyzed using commercially available colorimetric assays for the determination of BHB (MAK041, Sigma-Aldrich, St. Louis, MO), NEFA (SFA-5, Zen-Bio Inc., Durham, NC), glucose (TR15421, Thermo Fisher Scientific, Middletown, VA), and UN (EIABUN, Invitrogen, Middletown, VA) according to the manufacturer’s recommendations. Intra-assay coefficients of variation averaged 4.96, 4.46, 4.82, and 4.01%, and inter-assay coefficients of variation were 9.13, 6.66, 7.56, and 5.79% for the analysis of samples containing 0.19 mmol BHB/L, 3.78 mmol glucose/L, 0.14 mmol UN/L, and 0.06 mmol NEFA/L, respectively.

### 2.4 Doppler ultrasonography

Twenty-eight days post-breeding, pregnancy was confirmed using a linear transducer probe, and the corpus luteum was identified to determine the gravid uterine horn. Hemodynamic measurements of the uterine artery ipsilateral and contralateral to the corpus luteum were obtained via color Doppler ultrasonography (Sonosite Edge II; Fujifilm Sonosite Inc.; Bothell, WA) fitted with a 7.5-MHz linear transducer, as previously reported (Camacho et al., 2014). All measurements occurred within a 4-h preprandial period beginning 168 days post-breeding and repeated every 28 days throughout gestation. Three similar cardiac cycle waveforms from three separate ultrasonography evaluations for each side were obtained using spectral Doppler. The angle of insonation was recorded at less than 60° between the ultrasound beam and the uterine artery (Blanco, 2015). The maternal uterine artery peak systolic velocity (V_max_; cm/s), end-diastolic velocity (EDV; cm/s), resistive index (RI; unitless), ratio of peak systolic velocity to end-diastolic velocity (S/D; unitless), pulsatility index (PI; unitless), minimum diastolic velocity (MDV; cm/s), time-averaged peak velocity (TAP; cm/s), and diameter (cm), area (cm^2^), and circumference (cm) of the uterine artery ipsilateral and contralateral to the corpus luteum were calculated using preprogrammed Doppler software. The mean velocity (MV, cm/s) was calculated as MV = (V_max_ – MDV)/PI, and blood flow (BF, ml/min) was calculated as BF = MV × Area × 60. The total blood flow (TBF, ml/min) was calculated as the sum of ipsilateral and contralateral blood flow.

### 2.5 Statistical analyses

The Shapiro–Wilk test was used to assess the normality of the distribution of all variables. The main effects of maternal dietary treatment on the repeated measures of metabolites and hemodynamic parameters were determined using ANOVA with repeated measures using the MIXED procedure of SAS (SAS Institute Inc.; Cary, NC). The sources of variation for maternal metabolic parameters included the treatment, day, and their interaction. The sources of variation for maternal and neonatal metabolic parameters included the treatment, sample, and their interaction. The sources of variation for hemodynamic parameters included the treatment, day, side, treatment by day interaction, and treatment by side interaction. Day was used as the repeated variable, and the animal within the treatment was used as the subject. Covariates, such as maternal age and maternal body condition score, were tested to evaluate the fitness of the model. The main effect of the maternal dietary treatment on TBF was determined using ANOVA with repeated measures using the MIXED procedure of SAS with age and body condition score as the fixed effects. The sources of variation for TBF included the treatment, day, and their interaction. The least-square mean procedure was used to compare the means once significant differences were detected. Data are reported as the least-square mean ± SEM. Effects were considered significant when *p* ≤ 0.05.

## 3 Results

### 3.1 Metabolic profiles of dams subjected to divergent nutritional regimens and their offspring at birth

**FIGURE 1:**
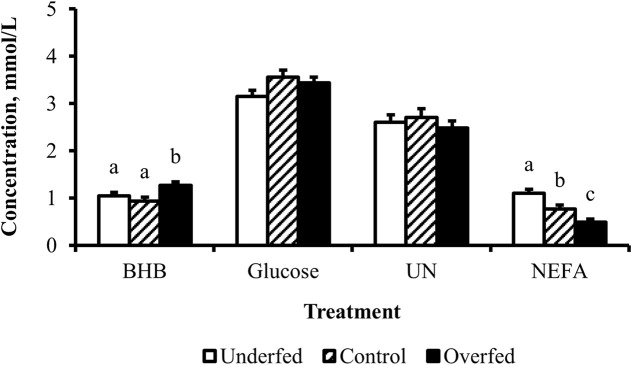
Maternal nutritional treatments applied throughout gestation induce changes in the concentrations of metabolites related to fat mobilization. Least-square mean + SEM (mmol/L) of BHB, glucose, UN, and NEFA in multiparous cows by maternal nutritional treatment. Data are pooled least-square mean for all time points throughout gestation. Maternal dietary treatments were Underfed, Control, and Overfed that provided 60%, 100%, and 140% of nutrient recommendations, respectively. Dietary treatments were offered from day 28 of gestation to parturition. Treatment *p*-values are equal to 0.02, 0.13, 0.63, and 0.0002 for BHB, glucose, UN, and NEFA, respectively. Treatments within metabolite with different lower-case letters are different (*p* ≤ 0.05).

**FIGURE 2:**
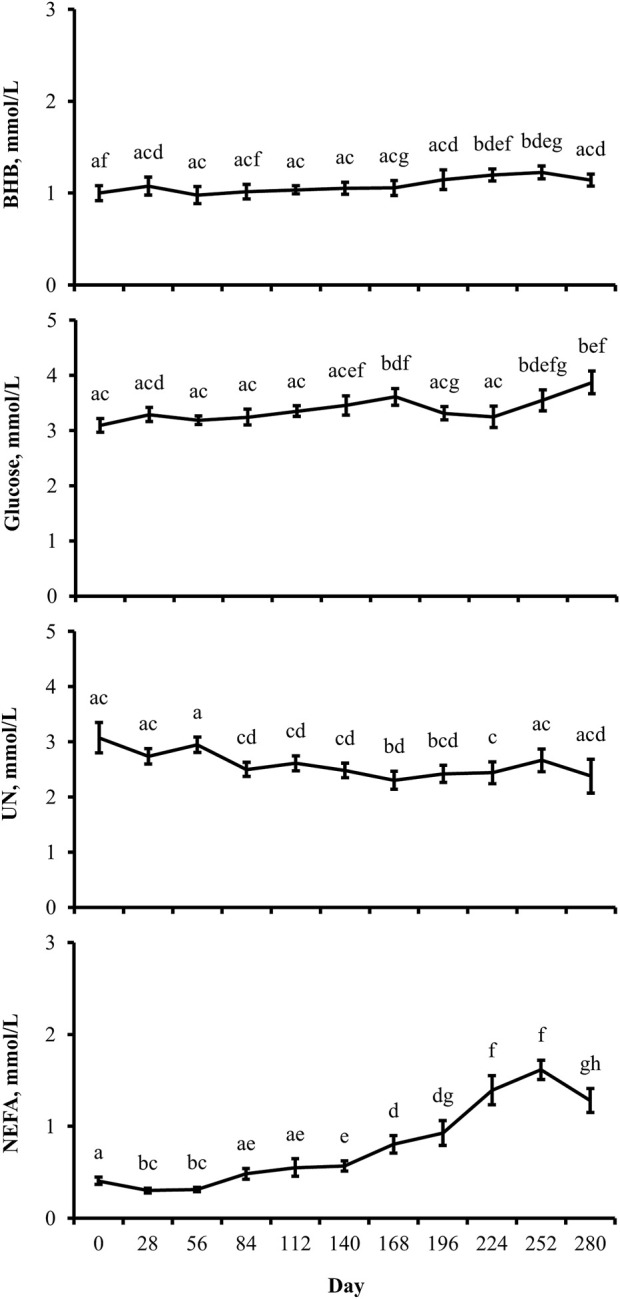
Distinctions exist between metabolite profiles during gestation, irrespective of the maternal nutritional treatment. Least-square mean ± SEM (mmol/L) of BHB, glucose, UN, and NEFA in multiparous cows by the day of gestation. Data are pooled least-square mean for all treatments throughout gestation. Maternal dietary treatments were Underfed, Control, and Overfed that provided 60%, 100%, and 140% of nutrient recommendations, respectively. Dietary treatments were offered from day 28 of gestation to parturition. Day *p*-values are equal to 0.05, 0.002, 0.02, and <0.0001 for BHB, glucose, UN, and NEFA, respectively. Days within metabolite with different lower-case letters are different (*p* ≤ 0.05).

**FIGURE 3:**
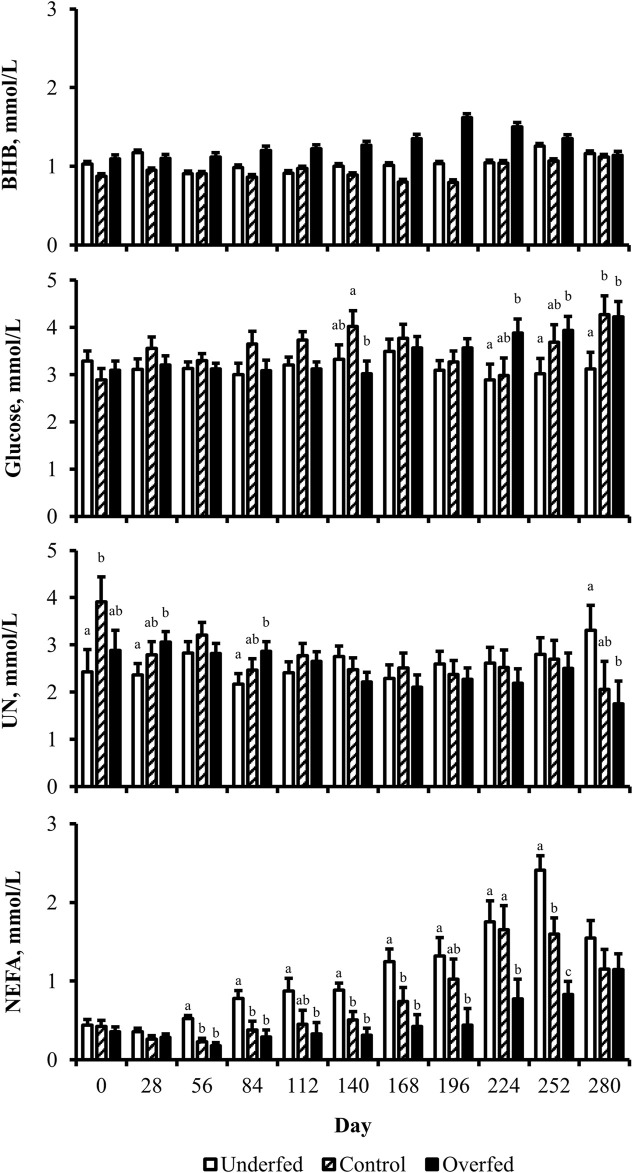
Maternal nutritional treatments induce changes in the concentration of metabolites during distinct periods of gestation. Least-square mean +SEM (mmol/L) of treatment by day interaction for BHB, glucose, UN, and NEFA in multiparous cows. Maternal dietary treatments were Underfed, Control, and Overfed that provided 60%, 100%, and 140% of nutrient recommendations, respectively. Dietary treatments were offered from day 28 of gestation to parturition. Treatment by day *p*-values are equal to 0.25, 0.02, 0.05, and 0.0002 for BHB, glucose, UN, and NEFA, respectively. Treatments within day with different lower-case letters are different (*p* ≤ 0.05).

**FIGURE 4:**
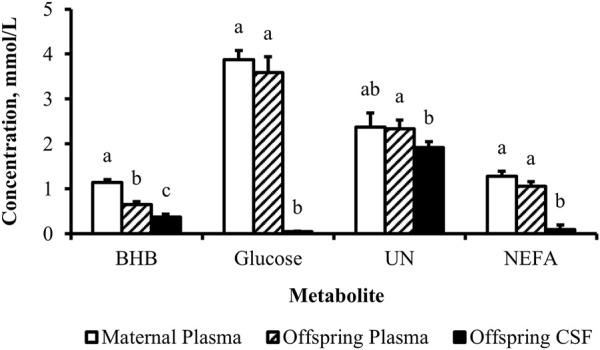
Concentration gradient from the maternal circulation to neonatal circulation to neonatal cerebrospinal fluid evident for metabolites. Least-square mean + SEM (mmol/L) by sample for BHB, glucose, UN, and NEFA in cow plasma, calf plasma, and calf cerebrospinal fluid at birth, pooled across treatments. Maternal dietary treatments were Underfed, Control, and Overfed that provided 60%, 100%, and 140% of nutrient recommendations, respectively. Maternal nutritional treatments were offered from day 28 of gestation to parturition. Sample *p*-values are equal to <0.0001, <0.0001, 0.009, and <0.0001 for BHB, glucose, UN, and NEFA, respectively. Samples within metabolite with different lower-case letters are different (*p* ≤ 0.05).

**FIGURE 5:**
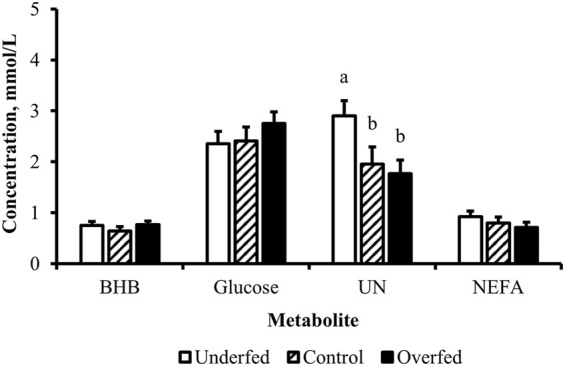
Influence of maternal nutritional treatment on the concentrations of metabolites at birth are limited to urea nitrogen. Least-square mean + SEM (mmol/L) by treatment for BHB, glucose, UN, and NEFA of samples pooled from cows and calves at birth. Maternal dietary treatments were Underfed, Control, and Overfed that provided 60%, 100%, and 140% of nutrient recommendations, respectively. Maternal nutritional treatments were offered from day 28 of gestation to parturition. Treatment *p*-values are equal to 0.49, 0.45, 0.04, and 0.39 for BHB, glucose, UN, and NEFA, respectively. Treatments within metabolite with different lower-case letters are different (*p* ≤ 0.05).

**FIGURE 6:**
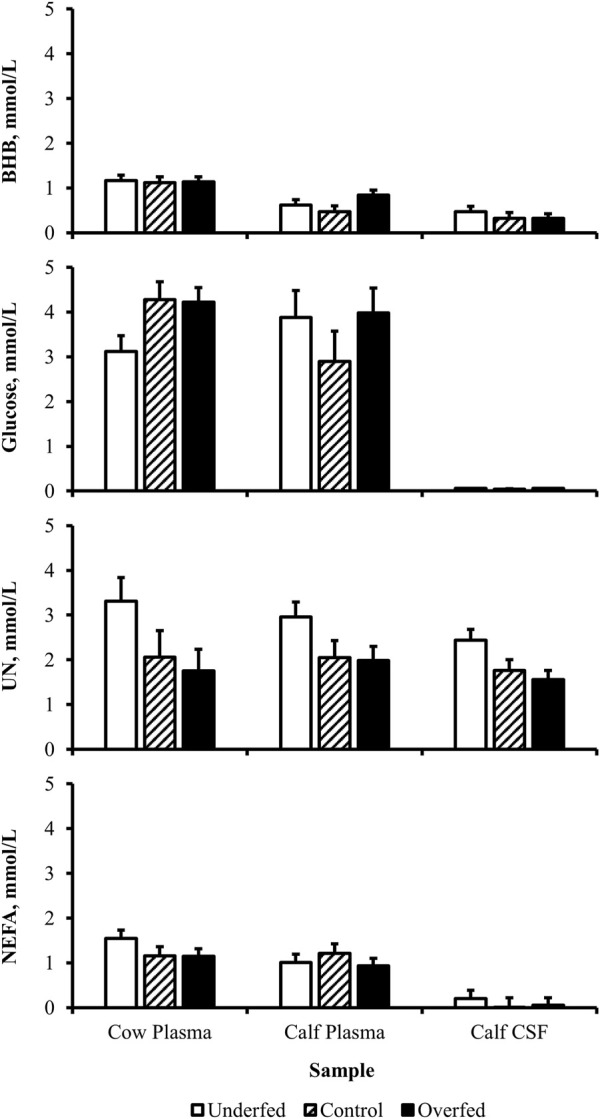
Maternal nutritional treatments fail to induce a change in the concentration of metabolites within samples. Least-square mean + SEM (mmol/L) of treatment by sample interaction for BHB, glucose, UN, and NEFA in cow plasma, calf plasma, and calf cerebrospinal fluid (CSF) at birth. Maternal dietary treatments were Underfed, Control, and Overfed that provided 60%, 100%, and 140% of nutrient recommendations, respectively. Dietary treatments were offered from day 28 of gestation to parturition. Treatment by sample *p*-values are equal to 0.27, 0.13, 0.73, and 0.60 for BHB, glucose, UN, and NEFA, respectively.

The concentration of BHB over the course of the experiment was elevated (*p* ≤ 0.05) in overfed cows compared to control and underfed cows (Figure 1). The concentration of BHB increased (*p* = 0.04) from day 0 to 252 and then returned to pretreatment values by day 280 (*p* = 0.18; Figure 2). The interaction of treatment by day for BHB was insignificant (*p* = 0.25; Figure 3). Comparison of the concentration of BHB in the dam and neonate revealed an influence of the sample (*p* < 0.0001) with a stepwise decrease in the concentration of BHB from maternal plasma to neonatal plasma to neonatal CSF (Figure 4). There was no influence of treatment (*p* = 0.49; Figure 5) or treatment by sample interaction (*p* = 0.27; Figure 6) on the concentration of BHB upon comparison from the dam to the neonate in the neonatal period.

The concentration of glucose over the course of the experiment did not differ (*p* = 0.13) by maternal dietary treatment (Figure 1). The concentration of glucose was influenced by the day of gestation (*p* = 0.002), with concentrations elevated on days 168, 252, and 280 compared to pretreatment values (Figure 2). An interaction of treatment by day (*p* = 0.02) was present, with differences between treatments occurring at mid- and late gestation (Figure 3). At mid-gestation, the concentration of glucose was reduced (*p* = 0.02) in overfed dams compared to control dams, with the concentration of glucose being intermediate in underfed dams. In late gestation, the concentrations of glucose were divergent between underfed and overfed dams (*p* ≤ 0.04). During the periparturient period, the concentration of glucose was reduced (*p* ≤ 0.03) in underfed dams compared to control and overfed dams. At birth, a significant gradient existed between the concentration of glucose in the plasma of the dam and neonate compared to the CSF of the calf (*p* < 0.0001; Figure 4). There was no influence of treatment (*p* = 0.45; Figure 5) or treatment by sample interaction (*p* = 0.13; Figure 6) on the concentration of glucose when comparing the dam to neonate during the neonatal period.

The concentration of UN over the course of the experiment did not differ (*p* = 0.63) by maternal dietary treatment (Figure 1). The concentration of UN was influenced by the day of gestation (*p* = 0.02), with concentrations reduced (*p* = 0.02) from day 0 to 168 (Figure 2). The concentration of UN then increased (*p* = 0.05) from day 168 to 252 and was not different (*p* = 0.10) from pretreatment values for the remainder of gestation. An interaction of treatment by day (*p* = 0.05) was present, with differences between treatments occurring at early and late gestation (Figure 3). At trial initiation, the concentration of UN was reduced (*p* = 0.04) in underfed dams compared to control dams, with overfed dams having an intermediate concentration (*p* ≤ 0.13). In early gestation, the concentrations of UN were reduced (*p* ≤ 0.04) in underfed dams compared to overfed dams, with control dams having an intermediate concentration (*p* ≥ 0.21) on days 28 and 84. In the periparturient period, the concentrations of UN differed between underfed and overfed dams (*p* = 0.03), with the concentration of UN in control dams being intermediate (*p* ≥ 0.12). At birth, the concentration of UN was lower (*p* = 0.01) in neonatal CSF than in neonatal plasma, while the concentration of UN in the dam was intermediate (*p* ≥ 0.10; Figure 4). Moreover, there was an effect of treatment (*p* = 0.04; Figure 5) on the concentration of UN, with the concentration of UN being higher (*p* ≤ 0.05) in samples collected at birth from underfed dams and their calves than in other treatments. There was no influence of treatment by sample interaction (*p* = 0.73; Figure 6) on the concentration of UN upon comparison from the dam to the neonate during the neonatal period.

The concentration of NEFA over the course of the experiment differed (*p* = 0.0002) by maternal dietary treatment, with concentrations of NEFA decreasing (*p* ≤ 0.03) from underfed to control to overfed dams (Figure 1). The concentration of NEFA was also influenced by the day of gestation (*p* < 0.0001; Figure 2). The concentration of NEFA initially decreased (*p* = 0.004) from pretrial to day 28, which was maintained (*p* = 0.75) through day 56. The concentration of NEFA then increased (*p* = 0.02) on day 140 and continued to increase (*p* < 0.0001) until day 252. The concentrations of NEFA decreased (*p* = 0.05) from day 252 to the periparturient period. An interaction of treatment by day (*p* = 0.0002) was present for the maternal concentration of NEFA (Figure 3). Differences (*p* ≤ 0.04) in the concentration of NEFA began early in gestation for underfed dams compared to control dams but were intermittently lost across gestation. Differences (*p* ≤ 0.03) in the concentration of NEFA between the control and overfed dams only occurred on days 224 and 252. At birth, a significant gradient existed between the concentration of NEFA in the plasma of the dam and neonate compared to the CSF of the calf (*p* < 0.0001; Figure 4). There was no influence of treatment (*p* = 0.39; Figure 5) or treatment by sample interaction (*p* = 0.60; Figure 6) on the concentration of NEFA upon comparison from the dam to the neonate in the neonatal period.

### 3.2 Hemodynamics of the uterine artery in dams subjected to divergent nutritional regimens

The influence of maternal nutrition throughout gestation on hemodynamic parameters was limited to measures of blood flow and total blood flow (Table 1). No other parameters were influenced by maternal nutrition (*p* ≥ 0.13). The blood flow and total blood flow were reduced (*p* = 0.005) in overfed dams compared to control dams, with blood flow in underfed dams being intermediate.

**TABLE 1:** Changes in hemodynamic parameters caused by maternal nutritional treatments applied throughout gestation are limited to blood flow and total blood flow, despite no differences in the hemodynamic parameters from which blood flow and total blood flow are derived. Least square mean ± SEM of uterine artery hemodynamic parameters in multiparous cows by maternal nutritional treatment^a^.

Parameter^b^	Treatment^c^			*p*-value
	Underfed	Control	Overfed	
V_max_, cm/s	115.30 ± 8.51	123.59 ± 9.30	108.52 ± 7.62	0.48
EDV, cm/s	59.64 ± 6.03	66.99 ± 6.59	58.31 ± 5.39	0.58
RI, unitless	0.56 ± 0.03	0.50 ± 0.03	0.57 ± 0.03	0.33
S/D, unitless	3.26 ± 0.57	2.47 ± 0.54	2.68 ± 0.46	0.59
PI, unitless	5.56 ± 2.62	0.90 ± 2.57	5.69 ± 2.17	0.34
MDV, cm/s	60.88 ± 5.65	65.28 ± 6.10	56.98 ± 5.01	0.59
TAP, cm/s	64.12 ± 5.67	72.77 ± 6.10	58.50 ± 5.01	0.23
Diameter, cm	0.82 ± 0.05	0.95 ± 0.05	0.81 ± 0.04	0.14
Area, cm^2^	0.43 ± 0.05	0.56 ± 0.05	0.42 ± 0.04	0.13
Circumference, cm	2.28 ± 0.14	2.58 ± 0.15	2.19 ± 0.12	0.18
MV, cm/s	61.44 ± 6.38	67.56 ± 6.83	54.50 ± 5.61	0.36
BF, ml/min	2,008.67 ± 237.98^ab^	2,631.17 ± 249.23^a^	1,516.62 ± 205.57^b^	0.02
TBF, ml/min	4,180.32 ± 516.66^ab^	5,757.63 ± 576.66^a^	3,203.51 ± 472.40^b^	0.02

^a^ Maternal dietary treatments were Underfed (n = 4), Control (n = 4), and Overfed (n = 6) that provided 60%, 100%, and 140% of nutrient recommendations, respectively. Dietary treatments were offered from day 28 of gestation to parturition.

^b^ V^max^, peak systolic velocity; EDV, end-diastolic velocity; RI, resistive index; S/D, peak systolic velocity to end-diastolic velocity ratio; PI, pulsatility index; MDV, minimum diastolic velocity; TAP, time-averaged peak velocity; MV, mean velocity; BF, blood flow; TBF, total blood flow.

^c^ Treatments within parameter with different lower-case letters are different.

In contrast to the influence of maternal nutrition, the day of gestation influenced all hemodynamic measures recorded, excluding the resistive index and the ratio of peak systolic velocity to end-diastolic velocity (*p* ≥ 0.12; Table 2). The peak systolic velocity increased (*p* < 0.001) from day 168 to 252 and was then maintained through day 280 (*p* = 0.38). The end-diastolic velocity did not differ (*p* ≥ 0.36) from day 168 to 224 but then increased (*p* = 0.01) in late gestation and was maintained until parturition (*p* = 0.52). The pulsatility index did not differ (*p* ≥ 0.58) from day 168 to 252 but then increased (*p* = 0.008) in the periparturient period. The minimum diastolic velocity increased (*p* = 0.007) from day 168 to 196 and remained elevated for the remainder of gestation (*p* ≥ 0.07). The time-averaged peak velocity increased (*p* < 0.0001) from day 168 to 252 and then decreased during the periparturient period to values no different from those on day 196 (*p* = 0.66). The uterine artery diameter, area, and circumference increased (*p* ≤ 0.0006) incrementally from day 168 to 280. The mean velocity increased (*p* = 0.006) from day 168 to 252 and then decreased to values no different from those on day 168 during the periparturient period (*p* = 0.21). The uterine artery blood flow and total blood flow increased (*p* ≤ 0.005) from day 168 to 196, which were maintained through day 224 (*p* ≥ 0.13). The uterine artery blood flow and total blood flow increased (*p* ≤ 0.02) further from day 224 to 252, which were maintained through day 280 (*p ≥* 0.55).

**TABLE 2:** Majority of hemodynamic parameters of the uterine artery change in response to the day of gestation. Least-square mean ± SEM of uterine artery hemodynamic parameters in multiparous cows by day of gestation.

Parameter^a^	Day^b^					*p*-value^c^
	168	196	224	252	280	
V_max_, cm/s	97.62 ± 5.82^a^	115.46 ± 5.98^b^	114.77 ± 5.68^b^	127.72 ± 5.77^cd^	123.45 ± 5.74^bd^	<0.0001
EDV, cm/s	55.61 ± 4.16^a^	59.07 ± 4.28^a^	58.74 ± 4.06^a^	68.55 ± 4.13^b^	66.2466 ± 4.10^b^	0.003
RI, unitless	0.47 ± 0.04	0.56 ± 0.04	0.54 ± 0.03	0.55 ± 0.04	0.6046 ± 0.04	0.12
SD, unitless	2.06 ± 0.73	2.46 ± 0.79	2.68 ± 0.69	4.30 ± 0.72	2.5238 ± 0.70	0.26
PI, unitless	1.17 ± 3.10^a^	3.48 ± 3.30^a^	1.03 ± 2.91^a^	1.42 ± 3.04^a^	13.16 ± 2.96^b^	0.02
MDV, cm/s	49.81 ± 4.16^a^	61.92 ± 4.31^bd^	58.91 ± 4.02^b^	65.05 ± 4.11^be^	69.5353 ± 4.08^de^	0.0003
TAP, cm/s	53.99 ± 4.27^a^	63.87 ± 4.43^b^	68.05 ± 4.11^bd^	73.87 ± 4.22^cde^	65.8681 ± 4.18^be^	0.0009
Diameter, cm	0.65 ± 0.03^a^	0.73 ± 0.03^b^	0.89 ± 0.03^c^	0.96 ± 0.03^d^	1.0475 ± 0.03^e^	<0.0001
Area, cm^2^	0.26 ± 0.03^a^	0.34 ± 0.03^b^	0.48 ± 0.03^c^	0.57 ± 0.03^d^	0.6719 ± 0.03^e^	<0.0001
Circumference, cm	1.77 ± 0.09^a^	2.02 ± 0.09^b^	2.45 ± 0.09^c^	2.64 ± 0.09^d^	2.8611 ± 0.09^e^	<0.0001
MV, cm/s	52.48 ± 4.93^a^	62.00 ± 5.13^ac^	64.76 ± 4.74^bcd^	67.55 ± 4.86^bce^	59.0496 ± 4.82^ade^	0.05
BF, ml/min	990.50 ± 204.71^a^	1736.76 ± 215.29^b^	2109.52 ± 194.63^b^	2680.61 ± 201.27^c^	2743.36 ± 198.48^c^	<0.0001
TBF, ml/min	2,175.95 ± 382.61^a^	3,862.65 ± 384.56^b^	4,361.77 ± 382.61^b^	5,633.49 ± 386.04^c^	5,868.58 ± 395.43^c^	<0.0001

^a^ V_max_, peak systolic velocity; EDV, end-diastolic velocity; RI, resistive index; S/D, peak systolic velocity to end-diastolic velocity ratio; PI, pulsatility index; MDV, minimum diastolic velocity; TAP, time-averaged peak velocity; MV, mean velocity; BF, blood flow; TBF, total blood flow.

^b^ Day within parameter with different lower-case letters are different.

^c^ Reported as overall, pooled *p*-values collected from underfed (n = 4), control (n = 4), and overfed (n = 6) dams.

All the recorded hemodynamic parameters, excluding the pulsatility index, were influenced by whether the measurement was obtained from the uterine artery ipsilateral or contralateral to the corpus luteum (side; Table 3). The pulsatility index was not influenced by the side (*p* = 0.23). The peak systolic velocity, end-diastolic velocity, minimum diastolic velocity, time-averaged peak velocity, diameter, area, circumference, mean velocity, and blood flow were greater (*p* < 0.0001) in measurements from the uterine artery ipsilateral to the corpus luteum. The resistive index and ratio of peak systolic velocity to end-diastolic velocity were greater (*p* ≤ 0.02) in measurements from the uterine artery contralateral to the corpus luteum.

**TABLE 3:** Pulsatility index, a common measure of fetal health, is the only unchanged hemodynamic parameter between sides. Least-square mean ± SEM of hemodynamic parameters from multiparous cows were obtained from the uterine artery ipsilateral and contralateral (side) to the corpus luteum.

Parameter^a^	Side		*p*-value^b^
	Contralateral	Ipsilateral	
V_max_, cm/s	81.97 ± 5.16	149.63 ± 5.11	<0.0001
EDV, cm/s	39.64 ± 3.67	83.65 ± 3.63	<0.0001
RI, unitless	0.61 ± 0.02	0.48 ± 0.02	0.001
S/D, unitless	3.71 ± 0.46	1.90 ± 0.44	0.02
PI, unitless	5.74 ± 1.99	2.36 ± 1.93	0.23
MDV, cm/s	38.73 ± 3.49	83.37 ± 3.45	<0.0001
TAP, cm/s	40.69 ± 3.53	89.57 ± 3.48	<0.0001
Diameter, cm	0.61 ± 0.03	1.11 ± 0.03	<0.0001
Area, cm^2^	0.22 ± 0.03	0.71 ± 0.03	<0.0001
Circumference, cm	1.66 ± 0.08	3.04 ± 0.08	<0.0001
MV, cm/s	36.78 ± 4.01	85.56 ± 3.94	<0.0001
BF, ml/min	490.67 ± 155.98	3,613.64 ± 151.87	<0.0001
TBF, ml/min	.	.	.

^a^ V_max_, peak systolic velocity; EDV, end-diastolic velocity; RI, resistive index; S/D, peak systolic velocity to end-diastolic velocity ratio; PI, pulsatility index; MDV, minimum diastolic velocity; TAP, time-averaged peak velocity; MV, mean velocity; BF, blood flow; TBF, total blood flow.

^b^ Reported as overall, pooled *p*-values collected from underfed (n = 4), control (n = 4), and overfed (n = 6) dams.

The interaction between the treatment and side influenced the diameter, circumference, and blood flow (*p* ≤ 0.04; Table 4). No other hemodynamic parameters recorded were influenced by the interaction between the treatment and side (*p* ≥ 0.08). The diameter was greater (*p* ≤ 0.0006) when recorded ipsilateral to the corpus luteum, irrespective of the treatment. However, the diameter recorded contralateral to the corpus luteum in underfed dams was reduced (*p* = 0.04) compared to control dams, while it was intermediate in overfed dams (*p* ≥ 0.07). The circumference was greater (*p* ≤ 0.0002) when recorded ipsilateral to the corpus luteum, irrespective of the treatment. The blood flow was reduced (*p* ≤ 0.0003) when recorded contralateral to the corpus luteum, irrespective of the treatment. Furthermore, the blood flow recorded ipsilateral to the corpus luteum was reduced (*p* = 0.009) in overfed dams compared to that recorded in control and underfed dams.

**TABLE 4:** Maternal overnutrition is detrimental to the blood flow of the uterine artery ipsilateral to the corpus luteum compared to ipsilateral measures in underfed and control dams. Least-square mean ± SEM of the interaction between hemodynamic parameters from multiparous cows were obtained from the uterine artery ipsilateral and contralateral (side) to the corpus luteum and treatment^a^.

Parameter^b^	Treatment by side^c^						*p*-value
	Underfed contralateral	Underfed ipsilateral	Control contralateral	Control ipsilateral	Overfed contralateral	Overfed ipsilateral	
V_max_, cm/s	81.00 ± 9.15	149.56 ± 8.83	87.41 ± 9.70	159.80 ± 9.71	76.63 ± 7.95	140.27 ± 7.99	0.38
EDV, cm/s	35.40 ± 6.48	83.29 ± 6.24	44.42 ± 6.84	89.55 ± 6.85	37.92 ± 5.61	78.57 ± 5.64	0.36
RI, unitless	0.67 ± 0.05	0.47 ± 0.04	0.56 ± 0.04	0.44 ± 0.04	0.61 ± 0.04	0.53 ± 0.04	0.35
S/D, unitless	4.98 ± 0.90	1.74 ± 0.79	3.02 ± 0.81	1.91 ± 0.82	3.35 ± 0.68	2.02 ± 0.71	0.45
PI, unitless	10.36 ± 3.85	1.57 ± 3.37	1.46 ± 3.51	0.40 ± 3.54	6.37 ± 2.94	4.99 ± 3.03	0.54
MDV, cm/s	34.67 ± 6.25	85.95 ± 5.93	45.01 ± 6.46	85.51 ± 6.47	35.34 ± 5.30	78.56 ± 5.35	0.25
TAP, cm/s	37.69 ± 6.35	90.12 ± 6.00	45.73 ± 6.52	99.85 ± 6.53	37.03 ± 5.36	79.75 ± 5.41	0.16
Diameter, cm	0.53 ± 0.05^ad^	1.10 ± 0.05^e^	0.71 ± 0.06^b^	1.19 ± 0.06^e^	0.57 ± 0.05^bd^	1.05 ± 0.05^e^	0.04
Area, cm^2^	0.16 ± 0.05	0.69 ± 0.05	0.31 ± 0.06	0.81 ± 0.06	0.19 ± 0.05	0.64 ± 0.05	0.18
Circumference, cm	1.48 ± 0.15^a^	3.07 ± 0.14^b^	1.94 ± 0.16^a^	3.21 ± 0.16^b^	1.55 ± 0.13^a^	2.83 ± 0.13^b^	0.03
MV, cm/s	34.9 ± 7.23	87.55 ± 6.80	38.83 ± 7.36	96.36 ± 7.38	34.41 ± 6.05	74.29 ± 6.11	0.08
BF, ml/min	293.51 ± 288.56^a^	3,723.83 ± 265.33^b^	746.68 ± 283.95^a^	4,515.65 ± 285.15^b^	431.80 ± 234.54^a^	2,601.43 ± 237.93^c^	0.002
TBF, ml/min	.	.	.	.	.	.	.

^a^ Maternal dietary treatments were Underfed (n = 4), Control (n = 4), and Overfed (n = 6) that provided 60%, 100%, and 140% of nutrient recommendations, respectively. Dietary treatments were offered from day 28 of gestation to parturition.

^b^ V_max_, peak systolic velocity; EDV, end-diastolic velocity; RI, resistive index; S/D, peak systolic velocity to end-diastolic velocity ratio; PI, pulsatility index; MDV, minimum diastolic velocity; TAP, time-averaged peak velocity; MV, mean velocity; BF, blood flow; TBF, total blood flow.

^c^ Treatment by side interactions within parameter with different lower-case letters are different.

## 4 Discussion

This is the first report that concurrently investigates the effect of under- and overnutrition throughout gestation on uterine artery hemodynamics. Furthermore, it is the first to report the metabolic profile of paired samples collected from the dam and neonate when dams are subjected to malnutrition throughout pregnancy. Moreover, this report concurrently assessed maternal uterine artery hemodynamics and metabolic profiles. These findings illustrate the complexity of the maternal-to-fetal interaction required to maintain the growth of the fetus and homeostasis of the dam.

The effect of maternal nutrition on uterine artery hemodynamics has previously been evaluated in cattle using supplemental protein, arginine, and dried distillers’ grains plus soluble; global nutrient restriction; nutritional realimentation; and heifer development management strategies (Camacho et al., 2014; Hernandez-Medrano et al., 2015; Yunta et al., 2015; Kennedy et al., 2016; Cain et al., 2017; Camacho et al., 2018; Lemley et al., 2018; Meneses et al., 2024; Redifer et al., 2024). However, no reports on bovine have assessed the influence of malnutrition, both underfed and overfed, throughout gestation on uterine artery hemodynamics. In an ovine model, other studies have demonstrated that maternal overnutrition reduced uterine vascularity (Wallace et al., 2002; Redmer et al., 2004). Similar to the current report, the uterine blood volume in the ewe lamb was reduced in response to maternal overnutrition. Of note, maternal undernutrition in bovine did not induce a reduction in uterine artery blood flow. This illustrates the commonality across species that fetal detriment is only observed in offspring from overfed dams.

A previous report utilized isocaloric diets of different crude protein contents offered to heifers during the periconception period (Hernandez-Medrano et al., 2015). In that report, the crude protein content failed to alter the blood flow volume. Similarly, others have reported that alterations in nutritional protein or amino acid status did not influence the blood flow volume (Yunta et al., 2015; Cain et al., 2017). However, when isonitrogenous diets of various energy statuses were offered in the current experiment, overnutrition reduced the blood flow volume. Therefore, it appears that the nutrient source is critical in inducing changes in the uterine artery blood flow.

In addition to the source of nutrients, the timing of the nutritional insult determines the extent of the impact on uterine artery blood flow. However, due to limitations in recording uterine artery parameters in the bovine, a majority of reports on the effects of maternal malnutrition on uterine artery hemodynamics have focused on the second half of gestation (Yunta et al., 2015; Kennedy et al., 2016; Cain et al., 2017; Lemley et al., 2018; Meneses et al., 2024; Redifer et al., 2024). Most reports that have assessed uterine artery hemodynamics in early gestation have not investigated the influence of maternal nutrition on hemodynamics (Ford and Chenault, 1981; Bollwein et al., 2002; Panarace et al., 2006). A report on early gestation has investigated the influence of arginine on uterine artery blood flow and revealed no influence of dietary treatment (Yunta et al., 2015). While findings vary across reports of various treatments applied across different periods, the mechanistic differences between these periods and how they may affect molecular metabolic mechanisms in the offspring are yet to be revealed.

The duration of the nutritional insult may also contribute to differences in hemodynamics. A variety of treatment durations have been utilized, but only two reports—including the current report—have assessed the extended application of maternal nutritional treatment on hemodynamic parameters (Wallace et al., 2002). The findings obtained by Wallace et al. (2002) align with those of the current report that maternal overnutrition reduces blood flow to the uterine artery. Interestingly, Wallace et al. (2002) utilized an ovine model, illustrating mechanistic conservation across species.

The current report utilized mature dams, while other reports investigated the influence of maternal malnutrition using immature dams (Wallace et al., 2002; Redmer et al., 2004; Yunta et al., 2015; Cain et al., 2017; Lemley et al., 2018). The use of immature dams compounds the demands of the growing dam with fetal development. We utilized a mature dam model in the current report to isolate the influence of maternal malnutrition while excluding maternal growth from the model. Additional reports have also revealed the influence of the breed of animal on blood flow parameters (Ferrell, 1991; Lemley et al., 2018). It is likely that differences in the breed are conserved across species and possibly contribute to differences between some reports.

Although it is critical to understand the ability of the dam to support fetal development, only a single previous report concurrently assessed uterine hemodynamics and circulating metabolites in the dam during gestation (Ferrell et al., 1982). Ferrell et al. (1982) reported glucose and urea concentration gradients across the uteroplacental unit during mid-gestation, while in the current report, glucose and urea concentration gradients existed across the blood–cerebrospinal fluid interface at birth. The stage of development influences fetal nutritional demand and nutrient preference. Therefore, it is likely that the day of gestation is a major contributor to the differences between the prior and current reports. Interestingly, a BHB concentration gradient from the dam to the neonate is present at birth in the current report. Moreover, the concentration of BHB was elevated in overfed dams. Beta-hydroxybutyrate plays a role in suppressing inflammasome activation in the placenta (Hirata et al., 2021), from which we infer that endogenous BHB production is upregulated in overfed dams in order to suppress malnutrition-induced inflammation.

A prior report assessed the metabolic profile of the cow throughout gestation (Prezotto and Thorson, 2022). The mean concentration of metabolites is comparable between control populations of the prior and current reports, excluding NEFA. The concentration of NEFA in the current report is approximately twice that of the prior report. Differences in reports are likely attributable to the population of cattle utilized. In the prior report, dams were a product of Angus cows and a line-bred Hereford sire. The linebred population of cattle is characterized by pronounced seasonal differences in adiposity, which likely contribute to the differences in NEFA concentrations between reports.

No previous reports have evaluated the influence of maternal nutritional treatment on the metabolite content in the blood and CSF of neonates. In the current report, we revealed that maternal undernutrition increased the concentration of urea nitrogen at birth. Elevated concentrations of urea nitrogen are associated with the disruption of the blood–brain barrier (Faucher et al., 2023; Bobot et al., 2024) and, therefore, have the potential to induce the aberrant development of nutrient-sensing nuclei within the central nervous system. This provides a potential physical mechanism programmed within the fetal blood–brain barrier via maternal malnutrition that has the potential to regulate offspring health and performance.

The population size of the current dataset is limited. However, the experimental design and methodology of the current report profoundly limit extraneous factors and, thus, allowed us to identify statistical differences attributed to nutritional status on hemodynamic and metabolic parameters. Each of the metabolites assessed in the current report crosses the placenta (Meschia et al., 1965; Seeds et al., 1980; Thomas, 1987; Kalhan and Parimi, 2000) and blood–brain barrier (Kleeman et al., 1962; Banks et al., 1997; Devraj et al., 2011; Shippy et al., 2020), thus creating a concentration gradient from the dam to fetal plasma and CSF. However, limited synthesis of glucose and UN can occur within the fetus and neonate, respectively (Gresham et al., 1972; Kalhan and Parimi, 2000). Moreover, the synthesis of BHB, UN, and NEFA can, to a limited degree, occur within the central nervous system (Buniatian and Davtian, 1966; Edmond et al., 1987; Aizawa et al., 2016). Therefore, it is possible that a portion of metabolites in the calf’s plasma and CSF originates from the peripheral and central systems of the offspring, in addition to the majority that originates from the dam.

Extended exposure to malnutrition requires metabolic plasticity as the animal shifts toward a catabolic state. Pregnancy adds additional stress on the dam to maintain homeostasis as she serves as a nutrient reservoir for the developing fetus. The current report reveals a novel avenue for investigating the influence of protracted maternal malnutrition on blood–brain barrier development and metabolic pathway preference in offspring. Tissues that serve as nutrient reservoirs, sense nutritional state, or regulate feeding behavior have a great influence on the health and productivity of offspring. Mechanisms that regulate the passage of nutrients and metabolites into the central nervous system likely contribute to the utilization of nutrients and life-long metabolic perception. Subsequent reports will reveal the profound influence of protracted maternal malnutrition on nutrient-sensing mechanisms within the central nervous system and the development of metabolic maladies.

## 5 Declaration of interest

Mention of trade names or commercial products in this publication is solely for the purpose of providing specific information and does not imply recommendation or endorsement by the USDA. The USDA prohibits discrimination in all its programs and activities on the basis of race, color, national origin, age, disability, and, where applicable, sex, marital status, familial status, parental status, religion, sexual orientation, genetic information, political beliefs, reprisal, or because all or part of an individual’s income is derived from any public assistance program (not all prohibited bases apply to all programs). Persons with disabilities who require alternative means for communication of program information (Braille, large print, audiotape, etc.) should contact the USDA’s TARGET Center at (202) 720-2600 (voice and TDD). To file a complaint of discrimination, write to the USDA, Director, Office of Civil Rights, 1400 Independence Avenue, S.W., Washington DC, 20250-9410, or call (800) 795-3272 (voice) or (202) 720-6382 (TDD). The USDA is an equal-opportunity provider and employer.

## Data Availability

The original contributions presented in the study are included in the article/Supplementary Material; further inquiries can be directed to the corresponding author.
